# Adipose Tissue-Derived Mediators in Multiple Myeloma: Linking Obesity to Bone Disease via Inflammatory Pathways

**DOI:** 10.3390/ijms26125618

**Published:** 2025-06-11

**Authors:** Alexandra-Ştefania Stroe-Ionescu, Alina Daniela Tǎnase, Ionela Rotaru, Janina-Georgiana Goanțǎ, Ana Maria Pǎtraşcu, Mihail Virgil Boldeanu, Mohamed-Zakaria Assani, Isabela Siloși, Lidia Boldeanu, Daniela-Teodora Maria

**Affiliations:** 1 Doctoral School, University of Medicine and Pharmacy of Craiova, 200349 Craiova, Romania; alexandra.stroe@umfcv.ro (A.-Ş.S.-I.); mohamed.assani@umfcv.ro (M.-Z.A.); 2 Department of Hematology and Bone Marrow Transplantation, Fundeni Clinical Institute Bucharest, 022328 Bucharest, Romania; alina.tanase@icfundeni.ro; 3 Department of Hematology, Faculty of Medicine, University of Medicine and Pharmacy of Craiova, 200349 Craiova, Romania; janina.goanta@umfcv.ro (J.-G.G.); anamaria.patrascu@umfcv.ro (A.M.P.); 4 Department of Immunology, Faculty of Medicine, University of Medicine and Pharmacy of Craiova, 200349 Craiova, Romania; isabela.silosi@umfcv.ro; 5 Department of Microbiology, Faculty of Medicine, University of Medicine and Pharmacy of Craiova, 200349 Craiova, Romania; lidia.boldeanu@umfcv.ro; 6 Department of Nephrology, Faculty of Medicine, University of Medicine and Pharmacy of Craiova, 200349 Craiova, Romania; daniela.maria@umfcv.ro

**Keywords:** multiple myeloma, bone disease, obesity, cytokines, adipokines, adiponectin, leptin, IGF-1

## Abstract

In patients diagnosed with multiple myeloma (MM), the primary complaints at the time of diagnosis are often related to bone involvement, significantly impacting quality of life and increasing both morbidity and mortality. Obesity is associated with a chronic inflammatory state that results in the production of various cytokines and adipokines, which may promote bone destruction. Adiponectin, an adipokine predominantly secreted by adipocytes, is notably diminished in circulation among individuals with obesity, a phenomenon that has also been observed in MM. This reduction may contribute to the disruption of an already compromised bone architecture. The increase in adipose tissue is associated with heightened leptin production, a key adipokine, which can play a significant role in the pathophysiology of MM and its related bone complications. Obesity is associated with hyperinsulinemia and increased levels of free IGF-1. In MM, IGF-1 plays a critical role as a growth factor, produced by both myeloma cells and osteoclasts within the bone marrow microenvironment. Our gathered data indicates a significant relationship between the adipokines produced by adipose tissue and the bone matrix, particularly in the context of obesity and MM. However, it is important to note that the existing body of research on this topic is relatively sparse, with the majority of studies conducted on murine models rather than human subjects. This limitation highlights a critical need for further investigation to elucidate the precise mechanisms that contribute to bone destruction under these conditions.

## 1. Introduction

Multiple myeloma (MM) is a malignant hematological condition characterized by the clonal expansion of plasma cells. It is defined by the secretion of monoclonal immunoglobulins and is associated with significant end-organ dysfunction, manifesting as hypercalcemia, renal impairment, cytopenias (particularly anemia), and osteolytic bone lesions [1,2,3]. Ranking as one of the most prevalent hematological malignancies after non-Hodgkin lymphomas, MM accounts for approximately 10% of these disorders [4,5]. Although it remains an incurable entity, advancements in therapeutic strategies, including the application of novel agents and high-dose chemotherapy followed by autologous stem cell transplantation, have markedly improved overall survival rates for affected patients [6,7,8].

The predominant concerns at the time of diagnosis are associated with bone involvement, which significantly impacts the patients’ quality of life and elevates morbidity and mortality rates. Approximately 80% of cases exhibit considerable skeletal lesions, characterized by complications such as fractures, chronic bone pain, diminished mobility, and spinal cord compression, often necessitating extended hospital stays for management [9,10,11]. Despite achieving a complete response in several cases, bone lesions continue to persist throughout the disease course. Notably, approximately 60% of patients are at risk of developing fractures [9,12,13]. Current guidelines endorse the use of whole-body, low-dose computed tomography (CT) or Fluorodeoxyglucose Positron Emission Tomography combined with CT (FDG-PET/CT) as the preferred imaging techniques for the diagnosis and evaluation of the extent of bone lesions. In terms of imaging diagnosis, these lesions may present as either extensive osteopenia or multiple lytic lesions, which can involve any skeletal region, though they are more commonly observed in the skull, spine, and ribs, affecting approximately 40–70% of cases [9].

Bone lesions result from a complex interplay of factors, predominantly driven by enhanced osteoclastogenesis coupled with suppressed osteoblast activity. This dysregulation arises from the interactions between myeloma cells and the bone microenvironment, facilitating osteoclast activation via several key signaling pathways, including the receptor activator of the nuclear factor-κB (RANK)/RANK ligand (RANKL) axis and Notch signaling [13,14]. This mechanism is further exacerbated by cytokines released within the bone marrow microenvironment, resulting from interactions between myeloma cells and bone marrow stem cells. Notable contributors include interleukin 6 (IL-6), IL-1β, and tumor necrosis factor-alpha (TNF-α) [13,14,15,16].

Recent research has increasingly highlighted the role of bone marrow adipose tissue (BMAT) in supporting myeloma cell survival and the progression of bone disease. Adipose tissue releases a range of cytokines and adipokines, which contribute to a state of chronic low-grade systemic inflammation, playing a pivotal role in inflammation-mediated bone loss [17,18,19]. Among these, adiponectin, an adipokine produced by adipose tissue, is known for its functions in glucose metabolism and insulin sensitivity, but it also exhibits pro-osteogenic properties by promoting osteoblast differentiation and inhibiting osteoclast activity. However, in obesity, adiponectin levels decline, leading to an upregulation of osteoclastogenesis and consequently enhancing bone resorption [20,21].

Obesity is a systemic condition that plays a significant role in the progression of monoclonal gammopathy of undetermined significance (MGUS) to MM, as well as in altering BMAT dynamics [22,23]. This review synthesizes the current literature to evaluate the interplay between obesity and bone lesions in MM patients, with a specific focus on the cytokines and adipokines associated with these conditions.

## 2. Obesity as a Risk Factor for Multiple Myeloma

Obesity has emerged as a significant public health crisis across the globe in recent years. As of 2022, approximately 890 million adults are grappling with obesity, a condition marked by excessive body fat that can lead to various health complications [24]. This troubling trend highlights the urgent need for awareness and intervention as it affects not only individual well-being but also healthcare systems worldwide. It is defined by the World Health Organization (WHO) as a chronic complex disease characterized by excessive fat deposits, with a Body Mass Index (BMI) ≥ 30 kg/m^2^, and further divided into three classes: class I (BMI 30.0–34.9), class II (BMI 35.0–39.9), and class III (BMI ≥ 40.0) [24]. Obesity is associated with a heightened risk of a variety of serious health conditions, including type 2 diabetes mellitus (T2DM) and heart disease. It also significantly contributes to the development of several types of cancer, such as colorectal cancer, renal cancer, postmenopausal breast cancer, prostate cancer, and leukemia [25]. Recent research has also focused on the correlation between obesity and an increased likelihood of developing MM, along with other plasma cell neoplasms. These findings highlight the critical health implications of obesity and its role as a major risk factor for multiple diseases. In 2016, the International Agency for Research on Cancer (IARC) identified MM as one of 13 malignancies positively correlated with obesity. Specifically, the relative risk of developing MM was quantified as 1.2 for individuals classified with class 1 obesity and 1.5 for those with class 2 or 3 obesity [26]. Additionally, findings from the PROMISE trial indicated that obesity is associated with a 73% increase in the odds of developing MGUS when compared to individuals with normal weight (odds ratio [OR], 1.73; 95% confidence interval [CI], 1.21–2.47; *p* = 0.003) [27]. MM is marked by skeletal-related events; however, the specific mechanisms by which obesity influences bone metabolism in this plasma cell neoplasm remain inadequately explored.

## 3. The Impact of Obesity on Bone Metabolism

Obesity is defined by an abnormal increase in adipose tissue, which results from both hyperplastic and hypertrophic changes in adipocytes [28,29]. Adipose tissue is increasingly acknowledged as a functional endocrine organ that synthesizes a variety of bioactive substances, including adipokines and diverse cytokines. These molecules play critical roles in mediating processes related to inflammation, immune responses, and the regulation of energy homeostasis [30,31,32,33]. In obesity, a persistent state of chronic inflammation is established, resulting in the secretion of pro-inflammatory cytokines such as IL-6, IL-1β, and TNF-α. These cytokines are recognized as key osteoclast-activating factors, contributing to the modulation of bone metabolism and potentially leading to an increased bone resorption activity [23,34]. A comparative study of obese TNF-α knockout mice and their wild-type counterparts indicated that the absence of TNF-α correlates with a reduced prevalence of osteoclasts and attenuated bone resorption. This finding suggests that TNF-α plays a significant role in the pathogenesis of bone metabolic disorders associated with high-fat diets [35,36]. IL-6 is a pivotal cytokine involved in bone metabolism and inflammatory responses. It is synthesized by various cell types, including adipocytes, and its production is notably influenced by the interaction between myeloma cells and bone marrow stem cells [37,38]. In a study examining MM cell lines co-cultured with adipocytes derived from patients across a spectrum of BMI categories—normal, overweight, obese, and super obese—it was found that higher BMI correlates with heightened IL-6 secretion. Specifically, IL-6 levels surged 3.8-fold in the overweight group, 4.6-fold in the obese cohort, and 4.1-fold in individuals classified as super obese, relative to adipocytes from normal-weight subjects [39]. To investigate the impact of IL-6 on obesity and bone metabolism, a study involving both wild-type and IL-6 knockout mice subjected to a high-fat diet was conducted. The findings revealed significant enhancements in trabecular bone metrics: IL-6-deficient mice exhibited a 53% increase in trabecular bone volume fraction, a 34% increase in trabecular number, and a 40% increase in trabecular thickness compared to their wild-type counterparts. These results indicate that IL-6 gene deficiency may mitigate bone loss associated with obesity [35,40].

IL-1β, a key pro-inflammatory cytokine typically found at elevated levels in obesity, plays a critical role in bone remodeling by enhancing osteoclastogenesis. It promotes bone resorption and extends the lifespan of osteoclasts through direct signaling mechanisms. Additionally, IL-1β stimulates the proliferation of osteoclast progenitors and their differentiation into mature osteoclasts [35,41,42]. Research involving obese mice on a 10% corn oil-based diet demonstrated significant increases in inflammatory cytokines, namely IL-1β, IL-6, and TNF-α. This inflammatory environment resulted in the upregulation of osteoclast-specific markers, including cathepsin K and RANKL, thereby favoring osteoclast formation and activity [35,43].

## 4. The Role of Bone Marrow Adipose Tissue in Myeloma Bone Disease

Among the various types of adipose tissue that play a significant role in the study of obesity, white adipose tissue (WAT) and brown adipose tissue (BAT) have been the subjects of extensive research. Recently, however, scientific inquiry has shifted toward a distinct category of adipose tissue, known as BMAT. This emerging player in the field of obesity research is gaining attention, as its unique characteristics and potential implications for metabolic health and weight regulation are beginning to be explored. Obesity is a significant contributor to an elevated BMAT, which in turn can adversely affect bone health. Research involving obese mice has demonstrated that this increased fat in the bone marrow results in a diminished recruitment of osteoblastic cells—key players in the process of bone formation. Consequently, the overall production of new bone tissue is reduced, highlighting the negative impact of obesity on skeletal integrity [44,45,46]. BMAT distinguishes itself from other adipose depots through its unique origin, dietary responsiveness, phenotype, gene expression, and physiological functions [17,28]. Its involvement in the pathogenesis of MM has garnered attention, particularly due to the intimate interactions between myeloma cells and the bone marrow microenvironment, highlighting a potential protective role of marrow adipocytes for these malignant cells. This protective effect is largely attributed to the BMAT’s capacity to secrete various chemokines, including monocyte chemotactic protein (MCP)-1 and stromal cell-derived factor (SDF)-1α, which facilitate the recruitment of myeloma cells, alongside other factors like hepatocyte growth factor, IL-6, and vascular endothelial growth factors that promote tumor cell survival and homing to bone niches [47,48]. Moreover, marrow adipocytes can activate autophagic pathways and upregulate autophagic proteins, thereby mitigating chemotherapy-induced apoptosis in myeloma cells [49]. The interplay of BMAT in bone metabolism within the context of MM remains an area of active investigation, with several hypotheses emerging. One notable theory implies that marrow adipocytes are capable of producing adipokines such as adiponectin, leptin, resistin, visfatin, and adipsin, which could modulate the differentiation of osteoclasts and osteoblasts [50]. Fluctuations in adipokine levels associated with obesity may exacerbate the growth, survival, and migratory capabilities of myeloma cells, along with contributing to bone destruction [51].

**Figure 1:**
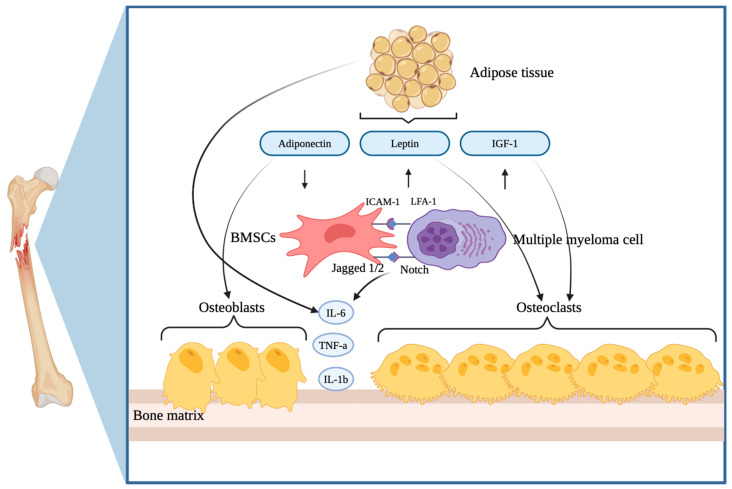
Schematic overview of different cytokines and adipokines produced by the adipose tissue and their influence on myeloma bone disease (Figure created using BioRender). The excess of adipose tissue leads to a decrease in adiponectin levels, which influences osteoblast activity, while leptin and insulin-like growth factor 1 (IGF-1) suffer a rise, leading to an increase in osteoclastogenesis. The interaction between bone marrow mesenchymal stem cells and MM cells promotes the production of IL-6, IL-1β, and TNF-α, which are further increased by the adipose tissue, leading to a pro-inflammatory state and enhanced osteolysis.

Conversely, a study analyzing bone marrow aspirates from newly diagnosed MM patients, those in remission, and healthy controls revealed that adipocytes from MM patients could induce osteolytic lesions by secreting factors that promote osteoclastogenesis and inhibit osteoblastogenesis, leading to net bone resorption. Quantitative polymerase chain reaction (PCR) analyses of various adipokines indicated the downregulation of adiponectin, adipsin, and visfatin, while the application of specific antibodies against these adipokines to osteoclast precursors suggested that they play a critical role in osteoclast differentiation [52]. The interplay between cytokines released from adipose tissue and the processes governing bone metabolism has been extensively examined in various studies (Figure 1). However, the impact of obesity on the development and progression of myeloma bone disease remains relatively underexplored. This gap in research highlights the critical need for further investigations to better understand how excess body weight may influence the pathophysiology of this condition and its effects on bone health.

### 4.1. The Influence of Adiponectin on Bone in Multiple Myeloma

Adiponectin, the predominant adipokine recognized for its anti-inflammatory properties, is primarily synthesized by adipose tissue. Additionally, it can be secreted by skeletal muscle, osteocytes, and lymphocytes, highlighting its diverse role in metabolic regulation and immune modulation [53,54,55]. It plays a critical role in the regulation of glucose and lipid metabolism, as well as in modulating insulin sensitivity. Additionally, it exhibits properties that are anti-inflammatory, anti-fibrotic, and antioxidant, contributing to metabolic homeostasis and the mitigation of oxidative stress [56,57,58]. Adiponectin appears to play a protective role in bone metabolism by promoting the proliferation and differentiation of osteoblasts through the adipoR1/JNK and adipoR1/p38 MAPK signaling pathways. Additionally, it inhibits osteoclastogenesis by downregulating key factors such as Macrophage Colony-Stimulating Factor (M-CSF) and RANKL [59,60]. A study involving adiponectin-deficient mice demonstrated a significant reduction in bone mass, characterized by an elevated density of osteoclasts both per bone perimeter and per bone surface. Additionally, there was an observed increase in the population of bone marrow adipocytes. The absence of adiponectin was associated with an elevated RANKL/OPG ratio in the bone marrow microenvironment, thereby enhancing the differentiation of osteoclasts [61]. A separate study revealed that the inhibitory action of adiponectin on osteoclasts is facilitated through the APPL1-mediated downregulation of Akt1 signaling activity [62].

Adiponectin, predominantly produced by adipocytes, has been observed to have significantly lower circulating levels in individuals with obesity compared to their lean counterparts [63,64,65]. This relationship extends to MM, as diminished serum adiponectin levels correlate with an increased risk of developing the disease [66,67]. Notably, a study focusing on MM patients revealed an inverse relationship between adiponectin levels and MM risk specifically among overweight and obese individuals, contrasting with findings in normal-weight subjects [68]. Furthermore, low adiponectin concentrations may facilitate the progression from MGUS to active MM [69]. While these findings underscore adiponectin’s role in MM pathophysiology, its influence on myeloma-induced bone disease requires further exploration. In a murine model, myeloma-bearing mice lacking adiponectin exhibited exacerbated osteolytic bone disease, characterized by reduced trabecular bone volume and impaired osteoblast function. Notably, administration of the apolipoprotein mimetic peptide (L-4F), recognized for its adiponectin-enhancing properties, resulted in a reduction in myeloma bone disease—evidenced by fewer osteolytic lesions and enhanced bone formation [70]. Liu et al. [71] conducted a study to elucidate the mechanisms by which adiponectin modulates osteoclast activity in MM. This study included 39 MM patients, from whom bone marrow and peripheral blood samples were collected. Bone disease classification was defined as follows: stage A indicated the absence of osteolytic lesions alongside the presence of osteoporosis; stage B involved the presence of one to three osteolytic lesions; and stage C was characterized by the existence of more than three osteolytic lesions and/or the occurrence of a pathological fracture. Patients with MM exhibited markedly reduced levels of adiponectin in their serum compared to healthy control subjects. Additionally, within the context of myeloma bone disease, adiponectin concentrations were significantly higher in both serum and bone marrow of patients classified in stage A of the disease. This finding contrasts sharply with the lower adiponectin levels reported in patients at stages B and C, suggesting a possible link between adiponectin levels and myeloma-related bone pathology. An examination of serum markers related to bone disease indicated a negative correlation between carboxy-terminal, cross-linking telopeptide of type I collagen (CTX) and adiponectin levels, while positive correlations were found with osteocalcin (OCN). The study further demonstrated that adiponectin downregulates the mTOR signaling pathway, as assessed by phosphorylation status of mTOR and its downstream signaling molecule, 4EBP1, subsequently inhibiting osteoclast differentiation and maturation [71].

In summary, the body of evidence supports the notion that adiponectin levels are altered in both obesity and MM, contributing to the disruption of already compromised bone architecture. However, the discrepancy between findings from murine models and clinical studies in MM patients highlights the pressing need for further investigations in this domain.

### 4.2. The Role of Leptin in Myeloma Bone Disease

Leptin, an adipokine, exhibits fluctuating serum levels in obesity in stark contrast to adiponectin. The accumulation of adipose tissue correlates with elevated leptin concentrations, which play crucial roles in modulating immune responses, angiogenesis, and lipolysis [72,73,74]. Additionally, leptin is implicated in bone metabolism, evidenced by its receptors present in primary osteoblasts and chondrocytes [75,76]. In murine models, the effects of leptin on osteoclast and osteoblast activity yield contradictory results. Research by Hamrick et al. [77], utilizing ob/ob mice (deficient in leptin) compared with lean controls, indicates that leptin deficiency adversely affects bone structure, evidenced by shorter femora, diminished femoral bone mineral content (BMC), reduced bone mineral density (BMD), and lower cortical thickness and trabecular bone volume. Interestingly, these leptin-deficient mice exhibited increased vertebral length, lumbar BMC, lumbar BMD, and trabecular bone volume. Bone marrow analysis from the femora of obese mice revealed a higher adipocyte count compared to normal mice [77]. Leptin appears to enhance the expression of pro-osteogenic factors within the bone marrow, thereby promoting bone formation. Intracerebroventricular and subcutaneous leptin administration in ob/ob mice resulted in increased BMD, BMC, and bone area, alongside a reduction in body weight, food intake, and body fat [78]. This suggests that leptin may offer protective effects on bone structure. However, other studies indicate that leptin may inhibit bone formation through a central nervous system-mediated pathway [79].

Leptin has garnered increasing attention in the area of cancer research. Numerous studies have highlighted its potential involvement in various neoplastic processes, impacting cancers such as colorectal, breast, prostate, non-small cell lung, and bladder cancer [80,81,82,83,84]. In particular, there is a remarkable association between leptin levels and the risk of MM. Research indicates that individuals diagnosed with MM exhibit significantly elevated concentrations of leptin in comparison to healthy individuals, suggesting a possible link between this adipokine and the progression of the disease [67,85,86].

Further investigations reveal that leptin can stimulate the proliferation of MM cells through the activation of critical signaling pathways, namely AKT and STAT3. This stimulation may compromise the effectiveness of chemotherapy treatments, posing a challenge in the therapeutic management of the disease [87]. Additionally, leptin is capable of enhancing the expression of BCL-2, a protein that helps cells resist programmed cell death, while also inhibiting the activity of caspase-3, thereby contributing to the development of resistance to apoptosis [87]. In a study conducted by Favreau and colleagues, the effects of leptin on anti-tumor immunity were explored within a murine model of MM. Their findings illustrated not only elevated levels of leptin and its receptor but also a disturbing interference with the anti-tumor functions of invariant natural killer T cells (iNKT), which are crucial for immune response [88]. In another investigation led by Liu et al., serum levels of both leptin and visfatin were quantified alongside several biomarkers indicative of bone disease, such as CTX, OCN, and procollagen I amino-terminal propeptide (PINP). Surprisingly, this study found no significant correlations between these bone disease markers and the levels of leptin or visfatin, highlighting an intriguing gap in our understanding [71]. Despite the comprehensive exploration of leptin’s role in the development of MM, a striking deficiency remains in our knowledge regarding myeloma-related bone disease. This void is particularly significant when considering the effects of leptin on intercellular adhesion molecule-1 (ICAM-1). ICAM-1 collaborates with RANKL in facilitating the formation of osteoclasts and the process of osteolysis, a phenomenon that has been notably observed in lung and breast cancers [89]. This unexplored avenue suggests that leptin may have more profound implications for bone health in the context of MM than currently understood.

### 4.3. The Role of IGF-1 in Myeloma Bone Disease

Obesity significantly contributes to the onset of metabolic disorders, including T2DM, hypertension, sleep apnea, and ultimately, cardiovascular pathology. The presence of obesity, coupled with metabolic syndrome, can result in hyperinsulinemia and elevated levels of free insulin-like growth factor 1 (IGF-1). This peptide hormone, predominantly synthesized in the liver in response to growth hormones, is integral to growth regulation and energy homeostasis [90,91,92], (Table 1). Emerging research indicate that IGF-1 may contribute to the pathogenesis of various malignancies, including prostate, breast, lung, and colorectal cancers, as well as certain hematological neoplasms. This peptide hormone appears to promote oncogenic processes by enhancing tumor proliferation and advancing disease progression [93,94]. In MM, IGF-1 is recognized as a key growth factor, synthesized by both myeloma cells and osteoclasts within the bone marrow microenvironment. Elevated levels of IGF-1 have been correlated with adverse clinical outcomes, indicating a poor prognosis for patients [95,96]. IGF-1 modulates multiple aspects of MM cell behavior, notably enhancing cell homing, proliferation, and angiogenesis. Studies using murine models have demonstrated that IGF-1 exhibits a chemotactic effect on 5T2 MM cells, primarily through the activation of the PI3K signaling pathway. Notably, these cells exhibit an increased expression of IGF-1 receptors on their surface, suggesting a heightened sensitivity to IGF-1 signaling in the context of myeloma progression [97]. The presence of IGF-1 enhances the proliferation rate of MM cells and promotes angiogenesis within the bone marrow by upregulating VEGF production via the MEK/ERK signaling pathway [98,99]. In the context of bone lesions characteristic of MM, Feliers and colleagues have shown that MM cells positioned near bone tissue secrete insulin-like growth factor binding protein-4 (IGFBP4). This secretion subsequently inhibits osteoblast proliferation and impairs bone formation [100]. Targeting the IGF-1 receptor with pharmacological agents such as picropodophyllin (PPP) has demonstrated notable efficacy in preclinical models. Treatment with these agents not only decreased tumor burden and angiogenesis but also alleviated osteolytic bone disease, resulting in enhanced survival rates in MM models. MicroCT analysis established the existence of osteolytic bone lesions in murine models harboring 5T2MM cells. Notably, treatment with PPP resulted in a significant reduction in the bone lesion index, returning it to levels comparable to the control groups. Additionally, in vitro osteoclast assays demonstrated that PPP treatment inhibited osteoclast differentiation in a dose-dependent manner, as evidenced by a lower mean count of TRAP+ cells in the treated group. This highlights the therapeutic potential of IGF-1R inhibition as a dual-action strategy that tackles both myeloma growth and the accompanying skeletal complications associated with the disease [101], (Table 2). Given the findings from IGF-1 knockout mice, which indicate that IGF-1 may play a role in regulating osteoclastogenesis by facilitating the differentiation of osteoclast precursors, a study focusing on glycosphingolipid GM3 was conducted [102]. Evidence suggests that IGF-1 plays a pivotal role in osteoclastogenesis within MM. IGF-1 acts synergistically with pro-osteoclastogenic factor RANKL to amplify osteoclast activation, significantly contributing to the development of osteolytic lesions in MM patients. Elevated levels of IGF-1 not only support tumor proliferation but also enhance the longevity and activity of osteoclasts, thereby worsening the extent of bone loss during disease progression. Inhibiting glycosphingolipid (GSL) biosynthesis has demonstrated efficacy in restraining osteoclast activation in MM. Notably, the glucosylceramide synthase inhibitor N-butyl-deoxynojirimycin (NB-DNJ) has been shown to disrupt the signaling pathways critical for osteoclastogenesis. This indicates that tumor-derived GSLs, along with the IGF-1 signaling pathway, play a significant role in modulating osteoclast activity. Therefore, targeting both GSLs and the IGF-1 pathway may present a promising dual-targeting strategy for therapeutic intervention in MM-related bone pathology [103]. However, studies on insulin-like growth factor binding protein-7 (IGFBP7) reveal its capacity to enhance osteogenesis in bone marrow stromal cells and counteract the inhibitory effects of activin A on osteoblast differentiation. This highlights the multifaceted role of IGFBP7 in bone metabolism, particularly in the context of MM. Notably, IGFBP7 administration resulted in a marked downregulation of DKK1, a key negative regulator of osteoblast function in MM. Additionally, IGFBP7 effectively neutralizes the inhibitory influence of activin A, which otherwise disrupts osteogenic processes. The activin A-mediated suppression of osteogenesis contributes to the disruption of the delicate equilibrium between bone formation and resorption, ultimately exacerbating myeloma-associated bone degradation [104].

The involvement of IGF-1 in myeloma bone disease remains inadequately understood, primarily due to the limited data available in the current literature. Existing studies indicate that IGF-1 may promote osteoclast activity, even though it is generally recognized as a bone anabolic agent. This paradox underscores the necessity for further research to elucidate the mechanisms by which IGF-1 influences bone remodeling in the context of myeloma.

## 5. Oxidative Stress and Its Role in Myeloma-Associated Bone Disease

Obesity is associated with elevated oxidative stress, which occurs when there is a dysregulation between the generation of reactive oxygen species (ROS) and the body’s antioxidant defenses. This imbalance can lead to cellular damage and contribute to the pathophysiology of obesity-related complications [105]. In MM, obesity-induced hyperglycemia, hyperlipidemia, and endothelial dysfunction contribute to elevated oxidative stress in the bone marrow microenvironment. This oxidative stress can compromise bone integrity and hinder the differentiation and function of osteoblasts, resulting in impaired bone remodeling and health [23]. ROS serve as downstream signaling molecules in the RANKL pathway and are implicated in the differentiation of osteoclasts [106,107]. The inhibition of ROS reduces osteoclast activity and osteoclastogenesis. Consequently, the depletion of nuclear factor erythroid 2-related factor 2 (NRF2), which normally functions to mitigate oxidative stress through its antioxidant mechanisms, results in an increase in intracellular ROS levels. This elevation in ROS ultimately promotes the differentiation and proliferation of osteoclasts, thereby enhancing osteoclastogenesis [108]. The production of ROS by immune cells in adipose tissue or bone marrow enhances osteoclast differentiation and subsequent bone resorption. This process is mediated through the upregulation of the nuclear factor of activated T cells, highlighting the intricate interplay between oxidative stress and bone metabolism [109]. However, given the sparse data currently available on this subject, there remains a pressing necessity for additional research to provide deeper insights.

## 6. Limitations and Future Directions

The main limitation is that a significant portion of the data originates from murine models. While these models are instrumental in elucidating biological mechanisms, their applicability to human physiology may be limited. There remains a pressing need for more comprehensive research, particularly involving studies focused on patients diagnosed with MM and obesity, to deepen our insights and improve treatment strategies. However, recent studies have introduced emerging players in myeloma-related bone disease, notably irisin, which shows potential therapeutic implications. Research indicates that irisin mitigates myeloma-induced trabecular bone damage by preserving femoral trabecular architecture, as evidenced by its effects on trabecular bone volume/total volume, trabecular number, trabecular fractal dimension, and enhancing trabecular separation in murine models of MM. Additionally, in cortical bone, irisin downregulates Sclerostin, a known inhibitor of bone formation, and RANKL, which promotes osteoclastogenesis. Concurrently, it enhances the expression of Osteoprotegerin, an anti-osteoclastogenic cytokine, within the bone marrow microenvironment [110]. These findings underscore the potential of irisin in playing a protective role in modulating bone metabolism in the context of myeloma.

## 7. Conclusions

The bone disease commonly associated with MM has been extensively studied, with a predominant focus on the immune landscape surrounding the condition. However, there is a notable gap in research exploring the potential interplay between obesity and the development of osteolytic lesions. Specifically, only a limited number of studies have sought to analyze the roles of certain cytokines and adipokines in this context, leaving an opportunity for deeper investigation into how these factors may influence the progression of bone disease in patients with MM. Obesity is already considered a risk factor for MM, but how it can influence bone metabolism still remains uncovered. Our gathered data indicates a significant relationship between the adipokines produced by adipose tissue and the bone matrix, particularly in the context of obesity and MM. However, it is important to note that the existing body of research on this topic is relatively sparse, with the majority of studies conducted on murine models rather than human subjects. This limitation highlights a critical need for further investigation to elucidate the precise mechanisms that contribute to bone destruction under these conditions. Understanding these pathways may pave the way for more effective interventions and treatments in the future. However, there seem to be promising results as new cytokines are investigated, such as irisin, which may play a role in the development of neoplasia and bone disease.

## Figures and Tables

**Table 1:** The impact of obesity on the concentrations of diverse adipokines and hormones.

Adipokine/Hormone	Effect of Obesity	Mechanism	Reference
Adiponectin	Decreased	Obesity suppresses adiponectin production; associated with insulin resistance.	[64]
Leptin	Increased	Elevated due to expanded fat mass; leads to leptin resistance.	[72]
IGF-1	Increased	IGF-1 levels may rise due to hyperinsulinemia.	[90]

Abbreviations: IGF-1: insulin-like growth factor-1.

**Table 2:** Correlation between adipokines/hormones, severity of bone disease, and markers of bone turnover in multiple myeloma.

Adipokine/Hormone	Severity of Bone Disease	Markers of Bone Turnover	Reference
Adiponectin	Lower levels of adiponectin were associated with three or more osteolytic lesions or pathological fractures.	Adiponectin levels were negatively correlated with CTX and positively with OCN.	[71]
Leptin	No significant correlations were found between leptin levels and bone disease.	The concentrations of leptin did not exhibit a significant correlation with serum biomarkers associated with bone disease.	[71]
IGF-1	Mice treated with PPP had a lower bone lesion index assessed by microCT.	The mean number of TRAP+ cells was higher in the vehicle group than in the PPP-treated group.	[101]

Abbreviations: CTX: carboxy-terminal cross-linking telopeptide of type I collagen; OCN: osteocalcin; IGF-1: insulin-like growth factor-1; PPP: picropodophyllin; TRAP+: tartrate-resistant acid phosphatase.

## Data Availability

The data used to support the findings of this study are available from the corresponding author upon reasonable request.
